# Toward Exotic Layered Materials: 2D Cuprous Iodide

**DOI:** 10.1002/adma.202106922

**Published:** 2022-01-19

**Authors:** Kimmo Mustonen, Christoph Hofer, Peter Kotrusz, Alexander Markevich, Martin Hulman, Clemens Mangler, Toma Susi, Timothy J. Pennycook, Karol Hricovini, Christine Richter, Jannik C. Meyer, Jani Kotakoski, Viera Skákalová

**Affiliations:** ^1^ Faculty of Physics University of Vienna Vienna 1090 Austria; ^2^ Eberhard Karls University of Tuebingen Institute for Applied Physics 72076 Tuebingen Germany; ^3^ NMI Natural and Medical Sciences Institute at the University of Tuebingen Markwiesenstr. 55 D‐72770 Reutlingen Germany; ^4^ University of Antwerp EMAT Antwerp 2020 Belgium; ^5^ Danubia NanoTech s.r.o. Bratislava Slovakia; ^6^ Institute of Electrical Engineering SAS Bratislava Slovakia; ^7^ Université Paris‐Saclay CEA CNRS LIDYL Gif‐sur‐Yvette 91191 France; ^8^ Laboratoire de Physique des Matériaux et Surfaces CY Cergy Paris Université Cergy‐Pontoise 95 031 France

**Keywords:** 2D materials, CuI, graphene encapsulation, heterostructures, layered materials

## Abstract

Heterostructures composed of 2D materials are already opening many new possibilities in such fields of technology as electronics and magnonics, but far more could be achieved if the number and diversity of 2D materials were increased. So far, only a few dozen 2D crystals have been extracted from materials that exhibit a layered phase in ambient conditions, omitting entirely the large number of layered materials that may exist at other temperatures and pressures. This work demonstrates how such structures can be stabilized in 2D van der Waals (vdw) stacks under room temperature via growing them directly in graphene encapsulation by using graphene oxide as the template material. Specifically, an ambient stable 2D structure of copper and iodine, a material that normally only occurs in layered form at elevated temperatures between 645 and 675 K, is produced. The results establish a simple route to the production of more exotic phases of materials that would otherwise be difficult or impossible to stabilize for experiments in ambient.

## Graphical abstract

Only a few dozen 2D crystals have been successfully prepared, though many more—including metal iodides—are predicted to exist. A major reason for this disparity is their instability under ambient conditions. A wet‐chemical method to synthesize 2D‐CuI, stabilizing it by graphene encapsulation in a one‐step process, is designed, and its chemical structure is imaged.

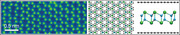

## 1 Introduction

Current 2D materials largely derive from van der Waals (vdW)‐layered bulk structures. However, only a limited number of such structures exist under ambient conditions and in total, only a few dozen 2D crystals have been successfully synthesized or exfoliated. While the unusual properties of graphene make it an interesting object of investigation itself,^[^ ^1^ ^]^ it can also serve as a substrate to stabilize other, less obvious 2D materials. These include materials that do not by themselves form 2D phases, such as the covalent SiO_2_,^[^ ^2^ ^]^ pseudo‐ionic PbI_2_,^[^ ^3^ ^]^ and metallic CuAu.^[^ ^4^ ^]^

In the same spirit, layers of graphene have also been used to encapsulate materials. Metal atoms (in some cases forming nitrides^[^ ^5^, ^6^ ^]^) have been intercalated between a monocrystalline SiC surface and graphene to produce 2D metamaterials.^[^ ^7^, ^8^, ^9^ ^]^ In other studies the encapsulation strategy has been applied in in situ transmission electron microscopy (TEM) observations of dynamics in liquids^[^ ^10^, ^11^ ^]^ and for protection of electron‐beam‐sensitive materials.^[^ ^12^, ^13^ ^]^ In addition, the inert and impermeable graphene envelope can also stabilize 2D layers of weakly bound molecules and atoms, and islands of C_60_ fullerenes^[^ ^14^ ^]^ and noble gases^[^ ^15^ ^]^ have been observed in graphene encapsulation. In the latter two examples especially, the significant, over 1 GPa (or 10^4^ atm) pressure associated with the vdW forces between graphene layers,^[^ ^16^ ^]^ is crucial to constraining the degrees of freedom and compelling the encapsulated species to assume and retain the 2D crystalline phase. The concept of encapsulation however is not limited to mere vdW materials and indeed, it entails a much greater variety of similarly constrained 2D structures that in their bulk form exhibit a vdW‐layered phase only either at elevated temperatures or pressures. One of such material among countless that have been predicted^[^ ^17^ ^]^ is the β‐phase of CuI that is only stable at a narrow temperature range of 645–675 K and therefore,^[^ ^18^, ^19^ ^]^ exfoliation of monolayers from such crystals would be rather complicated. The 3D γ‐phase of CuI, in contrast, is stable at ambient conditions and has been known for a high Seebeck coefficient (thermoelectrics) and appreciable optoelectric properties. Extremely thin layers of this nonlayered cubic γ‐phase of CuI have been successfully prepared and combined with 2D WS_2_ and WSe_2_.^[^ ^20^ ^]^ Nevertheless, the truly 2D phase of CuI, to our knowledge, has not yet been prepared and its properties remain unexplored. In fact, two distinct configurations of the 2D CuI structure were recently predicted by Mounet et al.,^[^ ^17^ ^]^ both unstable at ambient conditions; our primary motivation to this work therefore was to find out whether and which of the predicted structures could be synthesized.

Here, we demonstrate a method for the stabilization of a single layer of the high temperature vdW‐layered β‐CuI at ambient conditions by using graphene encapsulation. This new 2D material is synthesized directly between graphene layers in a single‐step wet‐chemical process and is henceforth called hexagonal copper iodide (2D h‐CuI). We fully characterize its atomic configuration experimentally and confirm the stability of the obtained heterostructure via density functional theory calculations. The experimental identification of the material is obtained through a combination of scanning TEM (STEM) atomic resolution *Z*‐contrast and ptychographic imaging, electron diffraction, X‐ray absorption spectroscopy (XAS), spatially resolved electron energy loss spectroscopy (EELS), as well as newly developed few‐tilt tomography. We believe that our results demonstrate a route to experimentally access further exotic 2D materials at room temperature.

## 2 Atomic Structure

**Figure 1:**
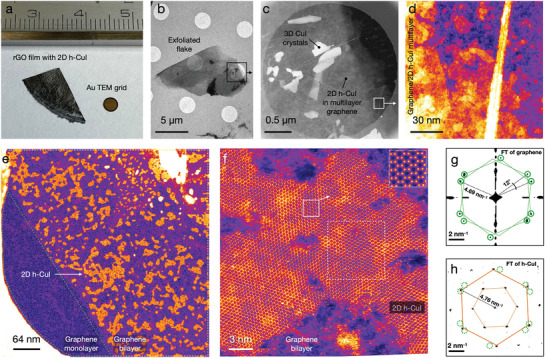
a) A film of reduced graphene oxide (rGO) incorporating 2D h‐CuI (the rule shows a centimeter scale). b) Bright‐field TEM image of a single graphene/h‐CuI flake suspended on a TEM support film. c,d) High‐angle annular dark‐field (HAADF) images of the flake edge. The orange and yellow contrast values in (d) are h‐CuI. e) HAADF overview image of monolayer h‐CuI crystals encapsulated in a bilayer graphene sandwich. No h‐CuI contrast is visible on the monolayer area on the left‐hand side. f) Atomically resolved HAADF closeup of a single 2D h‐CuI crystal with a magnifying inset in the top right corner. g) Frequency domain representation of graphene outside the crystal, and h) that of the area demarcated by the dashed square on the crystal in (f) is shown. The solid circles in (g) indicate the visible first‐order graphene reflections, superimposed on (h) using dashed circles. The images in (d–f) were false‐colored by applying the ImageJ lookup table Fire as an aid to the eye.

Macroscopic quantities of reduced graphene oxide (rGO) encapsulated 2D h‐CuI crystals were produced in film form as shown in **Figure** **1**a. The single‐step process is described in the Experimental Section. Note that due to the high quality of the rGO, the individual layers are practically indistinguishable from pure graphene. The layers that were manually exfoliated from the filtrated rGO/h‐CuI film and placed on a TEM grid for the STEM/TEM analysis had an average size of ca. 10–20 μm and a thickness that varied typically between one to ten layers. An example flake transferred onto a TEM support is displayed in Figure 1b. The flake edge area imaged in STEM high‐angle annular dark‐field (HAADF) mode (Figure 1c and d) show the layered nature of the structure that becomes progressively thinner toward its edge. Particularly, the crystallites that are inside the graphene bilayer in Figure 1e, display a uniform contrast over the entire field‐of‐view, implying they are of uniform thickness. Note that this image was selected because it shows both the monolayer and bilayer graphene areas of an exfoliated flake. Since the crystal islands are randomly dispersed only on the right‐hand side of the image on the area covered by the graphene bilayer and the monolayer part on the left is completely devoid of them, it is clear that the crystals are in graphene encapsulation. Additional example images, showing also larger h‐CuI domains and higher coverage, can be found in Figure S1, Supporting Information. The lateral size of the h‐CuI single‐crystal grains observed in the STEM/TEM images is up to 60 nm; nonetheless, the h‐CuI grains often form a covalently interconnected network which is extended to areas with a lateral size up to a micrometer. A closer inspection shown in Figure 1f reveals the crystals hexagonally symmetric lattice that matches to the expected symmetry of β‐CuI.

The periodicity of a lattice in the in‐plane direction can be analyzed by converting the projected real‐space image into the frequency domain via a Fourier transform, or alternatively by electron diffraction probing the reciprocal space directly. By selecting the particular areas of interest in Figure 1f, separate Fourier transforms were calculated for the graphene and the 2D h‐CuI, as displayed in Figure 1g,h. The period of the second‐order h‐CuI reflections matches closely the first‐order reflections of graphene, implying the commensurability of the lattices; for comparison, the graphene first‐order reflections (green circles) are indicated in both panels. The average value of the in‐plane lattice parameter of the 2D h‐CuI determined from electron diffraction is 4.19 ± 0.07 Å, which is slightly smaller than the 4.26 Å hexagon‐to‐hexagon distance along the armchair direction in graphene. Due to the possibility of a minor sample tilt adding a systematic error to the determined absolute values, the spacings were analyzed by directly comparing the graphene reflections with those of the 2D h‐CuI within individual nano‐beam diffraction images. It is also noteworthy that a slight anisotropy of 1–2% is observed with the nearest‐neighbor distances: in one direction the spacing is usually closely commensurate with the periodicity of graphene, whereas the distances in the perpendicular direction is slightly smaller. These minute differences are indicated in a nano‐beam electron diffraction pattern shown in Figure S2, Supporting Information.

The orientations of the graphene layers and the 2D h‐CuI crystals were also found to be highly correlated. In all instances, the orientations of the h‐CuI crystals match closely with one of the encapsulating graphene lattice directions with a 30° rotational translation, although small deviations from the ideal alignment are observable. For instance, the area highlighted with a rectangle in Figure 1f contains (besides h‐CuI) bilayer graphene with a layer mismatch angle of 12 ± 1°, as is indicated in the Fourier transform in Figure 1g. The graphene armchair edges in this particular case deviate by ca. 6° from the horizontal direction of the real‐space image, whereas the zigzag direction of the h‐CuI crystal deviates by ca. 2.5°. The edge of a cleaner 2D h‐CuI crystal is magnified in Figure S3, Supporting Information, showing also the crystal orientation with respect to graphene bilayer moiré. Note that the favored alignment is independent of the CuI crystallite size, but small crystals are sometimes seen oscillating between the two graphene orientations during image acquisition, indicating relatively weak interlayer binding. Even rotation angles of up to 30° are possible between two consecutive images. Examples are shown in Figure S8 and Video S1, Supporting Information.

## 3 Elemental Composition

**Figure 2:**
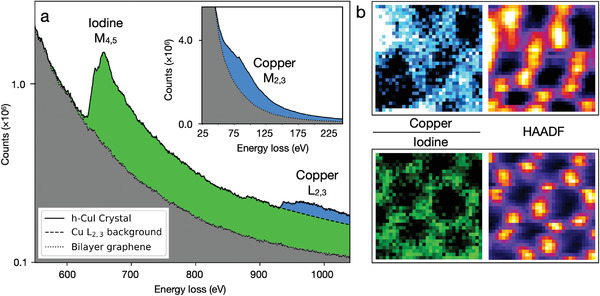
a) Iodine and copper electron energy core‐loss spectra recorded from a 2D h‐CuI crystal similar to the one shown in Figure 1f. The bilayer graphene reference was acquired from outside the crystal. The Cu *M*_2,3_ core‐loss edge is shown in the inset. b) Spatially resolved EELS maps showing Cu (integrated over 65–185 eV) and I (630–750 eV) distributions in the crystal. The distortion in the images is caused by the sample‐stage drift during the ca. 3 min of data acquisition needed to achieve the required signal‐to‐noise‐ratio. The greater variation on the Cu EELS map signal intensities is likely a result of lower electron‐beam stability of the Cu atomic sites. The field‐of‐view of the maps is 1×1 nm^2^. The right‐most panels show the concurrently acquired HAADF signals in false‐color (ImageJ lookup table Fire).

**Figure 3:**
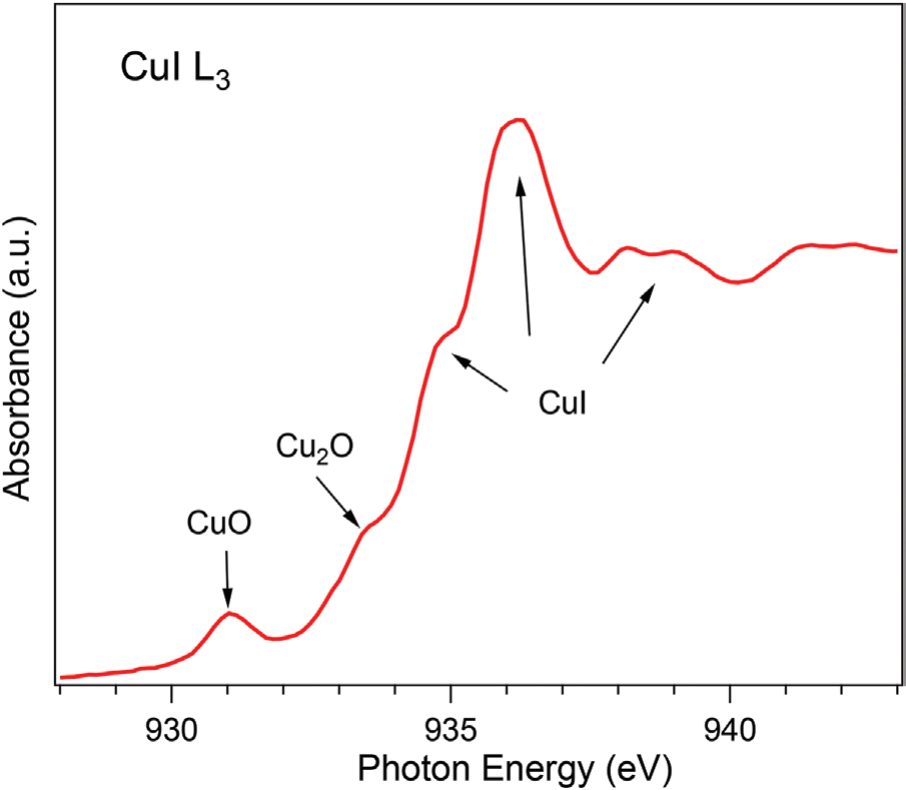
X‐ray absorption spectrum of CuI near the Cu *L*_3_‐edge. The arrows indicate different spectral features as explained in the text.

The elemental composition of the crystals was analyzed via EELS and the oxidation state of the elements via XAS. The results are summarized in **Figures** **2** and **3**. Figure 2a shows an energy‐loss spectrum acquired from a 4 × 4 nm^2^ area while continuously scanning the electron probe over a single h‐CuI crystal. The two core‐loss edges visible at 619 and 931 eV are associated with the I‐*M* and Cu‐*L* shell electron excitation. The pristine bilayer graphene spectrum (gray area) was recorded next to the h‐CuI crystal as a reference. The inset of Figure 2a shows the *M*_2,3_ core‐loss edge of copper. The spatial distributions of the core‐loss signal sources are presented as elemental maps in Figure 2b, and are well commensurate with the simultaneously acquired ADF‐signal. This result demonstrates that each of the apparent atomic positions is, in fact, occupied by at least a single I and Cu atom that are stacked exactly on top of one another as would be expected in β‐CuI.^[^ ^18^, ^19^ ^]^

The Cu *L*_3_ XAS spectrum shown in Figure 3 allows us to clearly identify the CuI compound thanks to several characteristic spectral features indicated by arrows, reproducing well the results from the literature.^[^ ^21^, ^22^ ^]^ The main absorption maximum at around 936 eV is assigned to the 2p → 4s (2p^6^3d^10^ → 2p^5^3d^10^4s^1^) transition combined with 3d–4s hybridization as detailed in ref. [21]. The shoulder close to 935 eV is part of the CuI characteristic line‐shape and has been attributed to a valence exciton.^[^ ^22^ ^]^

Two other features are present close to photon energies of 931 and 933 eV that we attribute to CuO and Cu_2_O compounds, respectively. Both copper oxides probably originate from the carbon tape, which contains them as we measured separately (the results are not shown here). As our CuI sample is a rather porous film, the signal from the supporting carbon tape can presumably add to the overall measured intensity. The white line of the iodine *M*_2_‐edge is certainly present as well but superimposed by CuO.

## 4 Atomic Structure in Cross Section

**Figure 4:**
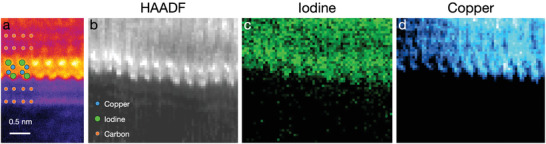
Cross‐sectional STEM images and EELS maps of the 2D h‐CuI structure within multilayer graphene encapsulation. a) Four‐time‐averaged STEM HAADF false‐color image (ImageJ lookup table fire) showing the graphene‐encapsulated 2D h‐CuI. b) STEM HAADF image obtained simultaneously to the elemental EELS maps. c,d) The iodine and copper *M*‐edge intensities. The iodine sites on the top row of the HAADF panels appear slightly brighter likely due to a greater overlap of the atoms on those atomic columns. The elemental EELS maps were produced by integrating the I‐*M* edge (630–750 eV) and Cu‐*M* edge (65–185 eV) signal intensities.

Three complementary techniques were further applied to study the 3D atomic structure: cross‐sectional imaging,^[^ ^23^ ^]^ observations of shallow‐angle tilted projections, including electron diffraction, and a recently developed method capable of reconstructing arbitrary 2D materials based on few‐tilt tomography.^[^ ^24^ ^]^ Cross‐sectional images provide the most direct proof of the atomic configuration and are presented in **Figure** **4**. Here, a folded graphene/h‐CuI heterostructure that includes several graphene layers crossing the image focal plane in profile was observed.

Figure 4a displays a high‐resolution HAADF image of a well‐resolved CuI layer between less visible layers of graphene: the top and bottom rows of bright atoms in the central 2D structure are likely iodine, matching the iodine‐terminating configuration for a monolayer of β‐CuI.^[^ ^17^, ^18^ ^]^ To visualize the distribution of iodine and copper atoms in the cross‐section, EELS maps were acquired by integrating iodine I‐*M* edge (630–750 eV) and copper Cu‐*M* edge (65–185 eV) intensities that are displayed in Figure 4c,d. A simultaneously acquired STEM HAADF image is shown in Figure 4b. Note that the EELS signals above the crystal's edge originates from the out‐of‐focus part of the bent flake, which is schematically depicted in Figure S4, Supporting Information. The fine‐structure of the crystal and even the I positions are still discernible in the middle part of panel (c). The distortion in the EELS maps, which is also visible in the simultaneously acquired HAADF image, results mainly from the sample stage drift during ca. 10 min of data acquisition, with a possible contribution from structural dynamics induced by the electron irradiation.

**Figure 5:**
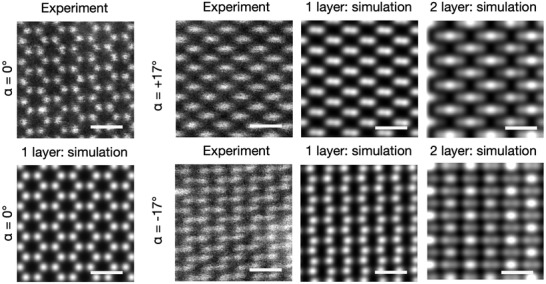
STEM‐HAADF projections of a h‐CuI crystal from different viewing angles shown with simulated views of a monolayer and AA‐stacked bilayer structures. The images in the left panels show the case of normal electron beam incidence where only the monolayer structure is considered (AA‐bilayer would display identical symmetry in the normal projection). The following frames compare the experimental and simulated projections along a single tilt axis α = ±17°. The image simulations are based on the density functional theory models shown in Figure 6 and Figure S10, Supporting Information. The bilayer out‐of‐plane unit cell length in the simulations was 7.345 Å. The scale bars in the images are 6.5 Å.

Additional evidence of the monolayer nature is obtained through observations of the structure from different viewing angles, with a single h‐CuI crystal being rotated around a pair of perpendicularly aligned tilt axes at ±17° angles. The resulting images are shown in **Figure** **5**, and a larger field of view of the area can be found in Figure S1, Supporting Information and images recorded for tilts along the second tilt axis are shown in Figure S5, Supporting Information. To interpret the experimental images, we simulated a number of views (see Experimental Section) with the same tilt angles as in the experiment and compared them with the experimental data. The simulated images are based on the optimized h‐CuI monolayer structure acquired via density functional theory (DFT) calculations discussed in more detail below. The bilayer image simulations were based on a DFT energy optimized β‐CuI structure with an out‐of‐plane unit cell length of 7.345 Å (see Table S1, Supporting Information). Whereas the simulated bilayer matches poorly with the experimental observations, the monolayer model is an excellent match in all projections. This result was further corroborated by electron diffraction tilt experiments that are discussed in Supporting Information (see especially Figure S7, Supporting Information).

With all evidence thus far pointing to a monolayer structure we now turn to the extraction of the 3D coordinates of Cu and I atoms within an individual h‐CuI layer. Few tilt tomography has previously enabled the 3D reconstruction of graphene with STEM ADF images from as few as two tilt angles.^[^ ^25^ ^]^ The CuI system however is far more challenging because it is not only several atomic layers thick, but also contains a mixture of heavy and light elements in each atomic column. Light elements are typically obscured in ADF images by the neighboring heavy atoms, as indeed also happens in the tilted projections here, and thus we complement the ADF signal with simultaneous single side band ptychography.^[^ ^26^ ^]^ Ptychography provides a greatly enhanced signal for a given electron dose compared to ADF imaging,^[^ ^27^ ^]^ and simultaneously resolves both heavy and light elements.^[^ ^28^ ^]^ Details of this analysis are given in the Experimental Section and in the Supporting Information. A more complete description of the method can be found in ref. [24].

**Figure 6:**
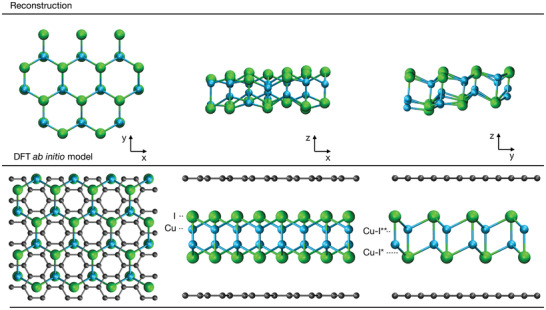
Reconstructed 2D h‐CuI crystal based on tilt experiments on the top row and an ab initio optimized structure in bilayer graphene encapsulation below it. The projections which the reconstruction is based on are shown in Figure S6, Supporting Information. The graphene layers in the DFT model are aligned in AA‐stacking. The DFT calculations are based on the optPBE‐vdW functional.^[^ ^30^ ^]^

**Figure** **6** shows a ball‐and‐stick model of 2D h‐CuI reconstructed from the experimental few tilt tomography together with an optimized structure obtained from the DFT calculations (see Experimental Section). Due to the convolution of the atomic positions with the atomic vibrations perpendicular to the plane, some uncertainty in the tomographic result is expected, especially in the out‐of‐plane direction.

With some minute differences, however, the experimental and computational models are in good agreement. For instance, the *z*‐displacement between the terminating iodine atomic planes in the ab initio model was 3.89 Å, whereas a separation of 3.42 ± 0.34 Å (mean and one standard deviation) was extracted from the reconstruction. The experimentally determined 1.63 ± 0.35 Å distance between the copper atomic planes, meanwhile, is slightly larger than the DFT result of 1.45 Å. The experimental Cu–I bond lengths were 2.67 ± 0.16 Å in the in‐plane direction and 2.55 ± 0.49 Å in the out‐of‐plane direction, whereas the DFT equivalents were 2.75 Å and 2.67 Å. These particular atomic bonds are also indicated in Figure 6 as Cu–I* and Cu–I**, respectively.

The DFT‐optimized separation of the graphene and CuI layers is 3.678 Å with a very small variation of ±0.013 Å depending on the interlayer stacking configuration (see Table S3, Supporting Information). In the most energetically favorable DFT configuration, iodine atoms are facing the centers of the graphene hexagons. The binding energies were calculated to be 14.1 ± 0.1 meV Å^−2^ per graphene layer, which is slightly higher than the calculated interlayer binding energy in bulk β‐CuI of 13.4 meV Å^−2^. A single layer of β‐CuI has been predicted to be a direct‐bandgap semiconductor.^[^ ^17^, ^29^ ^]^ Our calculations with the HSE06 hybrid functional give a bandgap of 3.17 eV for a single layer and 2.68 eV for bulk β‐CuI. Band structure calculations further suggest that in the CuI/graphene heterostructure, the characteristic linear dispersion of graphene π‐bands is preserved and the Dirac point appears in the bandgap of CuI (see Figure S13 and Table S2, Supporting Information).

## 5 Discussion

The structures of different cuprous iodide phases have in the past been subject to some level of controversy. Excluding the 2D phase presented here and the 1D structures observed in carbon nanotubes,^[^ ^31^ ^]^ a total of eight solid phases have thus far been identified.^[^ ^19^, ^32^ ^]^ The superionic phase occurring at high temperatures particularly follows from the chemical bonding of copper and iodine that is neither purely of covalent nor ionic nature, but exhibits both characteristics simultaneously.^[^ ^33^ ^]^ Normally, the semiconducting zinc blende bulk crystal^[^ ^34^ ^]^ is the primary phase in ambient conditions and it transforms into the layered β‐CuI phase at ca. 645–647 K,^[^ ^18^, ^19^, ^32^ ^]^ and which we presently obtained also at room temperature in 2D form by graphene encapsulation. The aforementioned controversy emerged in 1952 when the vdW‐layered β‐CuI phase was simultaneously but erroneously identified as a wurtzite structure by two independent research groups.^[^ ^35^, ^36^ ^]^ This error was pointed out by Bührer and Hälg in 1977,^[^ ^37^ ^]^ but since the authors did not provide the correct unit cell description, the confusion remained and the wurtzite structure sometimes resurfaces in the scientific literature.^[^ ^38^ ^]^ However, first‐principle calculations imply that the wurtzite phase might, after all, exist near the ground state.^[^ ^39^ ^]^ To our knowledge, the β‐CuI phase was first described by Sakuma in 1988^[^ ^18^ ^]^ and only later confirmed by Keen and Hull,^[^ ^19^ ^]^ and Sakuma should be rightly credited for this contribution.

The size of the 2D single crystal domains we observe span from a few nanometers to some tens of nanometers and exhibit high stability under 60 keV electron‐beam exposure in ultrahigh vacuum conditions. Although interconnected islands often cover areas as large as micrometers across, significant intrinsic disorder in the form of grain boundaries and nanopores is typically observed in them (see, e.g., Figure S1, Supporting Information). We believe that the crystal size might be limited by the slight incommensurability of the graphene and h‐CuI lattices, reflected in the measured anisotropy for the different lattice directions. This likely results in a small but significant strain energy penalty that can be sustained only for a finite crystal domain size, as well as through the appearance of nanopores and voids. The in‐plane lattice constant of the 2D h‐CuI crystal measured here is also somewhat smaller than what was previously reported for the bulk crystals. Sakuma measured a value of 4.279 Å for the β‐CuI phase at 693 K using X‐rays^[^ ^18^ ^]^ and Keen and Hull 4.304 Å at 655 K in neutron diffraction experiments,^[^ ^19^ ^]^ whereas we measured 4.19 ± 0.07 Å for a monolayer by using nano‐beam electron diffraction at room temperature. This apparent discrepancy might be due to thermal expansion at temperatures normally required to stabilize the β‐CuI phase (a spacing of ca. 4.21 Å at room temperature can be extrapolated based on the thermal expansion coefficient defined by Keen and Hull^[^ ^19^ ^]^). This value also matches our DFT calculations that predict a 4.20 Å spacing for both a monolayer and a bulk crystal without the graphene envelope (see Figure S10 and Table S1, Supporting Information). As was also pointed out by Sakuma,^[^ ^18^ ^]^ each atom in this particular crystal structure has exactly three neighbors and the crystal belongs to the $P\bar{3}m1$ Hermann Mauguin space group. To our knowledge, the h‐CuI crystal is one of few 1:1 stoichiometric 2D materials within this particular symmetry (hexagonal boron nitride being the most well known), and, besides PbI_2_,^[^ ^3^ ^]^ the only one that incorporates also a halogen atom.

The discovery of 2D h‐CuI is particularly interesting, since magnons^[^ ^40^ ^]^ and layer‐structure‐dependent ferromagnetism have recently been reported in CrI_3_,^[^ ^41^, ^42^, ^43^ ^]^ and a host of further magnetic ordering phenomena have been predicted in other 2D metal‐halides such as in NiI_2_ and CoI_2_.^[^ ^17^, ^29^, ^44^, ^45^ ^]^ Although DFT simulations have suggested the possibility of exfoliating some of those compounds, including 2D h‐CuI,^[^ ^17^ ^]^ their thermodynamic stability under ambient conditions remains untested. Indeed, based on the experiments by Sakuma^[^ ^18^ ^]^ and Keen and Hull,^[^ ^19^ ^]^ it seems obvious that 2D h‐CuI would not be stable at room temperature without encapsulation. The stabilizing function of graphene encapsulation was also manifested in our high‐resolution TEM experiments: after 80 keV electrons breached one of the graphene layers, the 2D h‐CuI rapidly broke apart (see Figures S8 and  S9; Video S1, Supporting Information).

Finally, there is no reason to believe that the present synthesis approach, which was here used to stabilize crystals of 2D cuprous iodide for room temperature experiments, would be limited to this particular structure. Quite the contrary, we have already applied the same process to produce 2D silver iodide (AgI) and nickel iodide (NiI_2_) crystals, both of which are shown in Figure S14, Supporting Information, and generalizing this concept should allow access to further exotic layered structures vastly expanding the currently available library of 2D materials, and their incorporation in devices and their structure and properties to be studied at room temperature.

## 6 Experimental Section

### Sample Preparation

The synthetic route to 2D h‐CuI confined between layers of graphene based on a wet chemical process was developed by Danubia NanoTech s.r.o. in Bratislava. First, GO was produced by the modified Hummers’ method^[^ ^46^ ^]^ resulting in 95% of single atom thick GO flakes with a lateral size 5–25 μm. Then 10 mL water dispersion of GO at a concentration of 1.5 mg mL^−1^ was vacuum‐filtrated through a polycarbonate (PC) membrane. Then, a cooled solution (0 °C) of 20 mg copper chloride (CuCl_2_) in 2 mL water was added. The last step was reduction of GO adding 1 mL of hydroiodic acid (HI). HI plays a double role: 1) H^+^ reduced the oxide groups from GO forming H_2_O whereas 2) I^−^ anions reacted with Cu^2 +^ cations forming the novel 2D crystal structure, h‐CuI. The crucial aspect was timing of the processes: CuI was formed during reduction when the interlayer distance of GO flakes  0.8 nm collapsed to 0.35 nm, the interlayer distance of the rGO planes; consequently, graphene sheets tightly wrapped the 2D h‐CuI crystals. The applied pressure of van der Waals force kept the 2D h‐CuI stabilized. The resulting layer was rinsed by ethanol, separated from the PC membrane, and dried. Even though, between the graphene layers exclusively 2D CuI is observed, it was difficult to avoid the formation of 3D CuI on the outer (farther from filter) surface of rGO film. Therefore, in all experiments, the surface of the filtrated film was removed by exfoliation with adhesive tape. For electron microscopy, reduced graphene oxide flakes produced by the chemical process were exfoliated under an optical microscope with the micromechanical cleavage method using adhesive tape. Flakes of a few layers in thickness were adhered onto a TEM grid by pressing the exfoliated material attached on the tape against the grid. Standard Au TEM grids with 300 mesh and Quantifoil amorphous carbon support were used. For some samples, to increase mechanical stability of small flakes, same grids with pretransferred monolayer graphene were used.

### (Scanning) Transmission Electron Microscopy

Scanning transmission electron microscopy experiments were conducted using an aberration corrected Nion UltraSTEM 100 microscope in Vienna, operated at 60 keV electron energy, with the sample in ultrahigh vacuum conditions at a pressure of 10^−10^ mbar. The angular range of the HAADF used for the image acquisition was 80–300 mrad. The electron energy loss spectra were recorded with a combination of Gatan PEELS 666 spectrometer and an Andor iXon 897 electron‐multiplying charge‐coupled device camera with the same instrument. The energy dispersion used in the experiments was 0.5 eV/pixel. The spectral maps in Figure 2 were acquired with a pixel dwell time of 150 ms and comprised 32 × 32 pixel arrays, whereas the maps in Figure 4 comprised 64 × 64 pixel arrays. The integrated core‐loss peak intensities for the spectral maps were produced by fitting a power‐law background for each peak separately and integrating over the given energy range.

The nano‐beam and parallel beam electron diffraction experiments were conducted using an image‐corrected JEOL ARM 200F HR‐TEM operated at 80 keV electron energy. The nano‐beam diffraction data was acquired using a probe size of 5–10 nm with beam convergence of ca. 2 mrad, allowing a high spatial selectivity.

Microsecond‐dwell‐time electron ptychography was conducted using a fast, event‐driven timepix3^[^ ^47^, ^48^ ^]^ camera in a probe‐corrected FEI ThemisZ instrument with a probe convergence angle of 30 mrad and a beam current of ca. 1 pA. Diffraction patterns as a function of probe position (also referred to as 4D‐STEM) were recorded simultaneously with the HAADF signal.

### TEM/STEM/Electron Diffraction Simulations

STEM image simulation were carried out with the multislice PyQSTEM Package.^[^ ^49^ ^]^ The simulation parameters were chosen to be the same as in the experimental data and the source size was chosen in order to have a similar broadening as in the experimental images. The number of slices was four for the normal incident electron beam and was increased to 30 when the model was tilted. Diffraction patterns were simulated by first carrying out a TEM simulation and by squaring the exit wave in the diffraction plane.

### X‐ray Absorption Spectroscopy

The absorption spectrum of the Cu *L*_2/3_‐edge was recorded at the BACH beam‐line of ELETTRA synchrotron light source in the total‐electron‐yield mode, where a drain current was measured from the sample fixed on a carbon adhesive tape. For the accurate determination of the photon energy the photo emission spectrum Au(4f) was acquired from a gold‐foil fixed on the sample plate. The BACH beamline works in the extreme‐UV–soft X‐ray photon energy range (35–1650 eV) with selectable light polarization, high energy resolution, and high intensity and brilliance. The sample environment was completely in ultrahigh vacuum.

### 3D Reconstruction

The complete 4D data set was processed to extract the phase of the electron wave by single side‐band (SSB) ptychography. The resulting phase images were used to increase the amount of information in the tilted projections to facilitate the visualization of lighter elements.^[^ ^28^ ^]^ The 3D reconstruction method is based on an optimization process where the SSB and ADF simulations of a model were matched to the whole experimental data set, including all different tilt angles. Details of the theoretical framework, discussions of the accuracy and comparison with only ADF‐based reconstructions can be found in ref. [24].

### DFT Calculations

All atomistic simulations were performed using DFT as implemented in the GPAW package.^[^ ^50^ ^]^ The core electrons were described using projector augmented wave potentials.^[^ ^51^ ^]^ The wave functions were expanded in a plane‐wave basis set with an energy cutoff of 650 eV. The optPBE‐vdW density functional^[^ ^30^ ^]^ was used to describe exchange and correlation as well as van der Waals interactions. Sampling of the Brillouin zone was performed according to the Monkhorst–Pack scheme^[^ ^52^ ^]^ using Γ‐centered 12 × 12 × 9 and 12 × 12 × 1 *k*‐point grids for unit cells of bulk and single layer β‐CuI, respectively. For the unit cell of the h‐CuI/graphene heterostructure, that has a size of 3 × 3 supercell of graphene, 9 × 9 × 1 *k*‐point grid was used. The h‐CuI/graphene unit cell is shown in Figure S12, Supporting Information. A vacuum of 20 Å was included to separate slabs in the *z*‐direction. The structures were optimized until the force on any atom was less than 10 meV Å^−2^. Due to the small incommunsurability between the graphene and h‐CuI lattices, a strain of ca. 1.6% was applied to the h‐CuI crystal in the calculations that included graphene, expanding the lattice constant of h‐CuI from 4.20 to 4.27 Å and allowing periodic boundary conditions. To calculate the electronic bandgap of CuI the Heyd–Scuseria–Ernzerhof (HSE06) hybrid DFT functional^[^ ^53^ ^]^ was used.

## Conflict of Interest

The authors declare no conflict of interest.

## Author Contributions

P.K., V.S., and M.H. developed the method and synthesized the 2D h‐CuI films encapsulated between graphene layers. K.M. prepared the samples for electron microscopy and conducted the STEM, whereas C.H. and J.C.M performed the HR‐TEM and nano‐area electron diffraction experiments. C.H. and T.J.P. used SSB ptychography method for 3D analysis of the 2D h‐CuI structure. The DFT calculations were done by A.M. The results were analyzed by K.M., C.H., A.M., and V.S. with input from J.K., T.J.P., J.C.M., and T.S. The manuscript was written by K.M., C.H., J.K., T.J.P., and V.S. with contributions from all co‐authors.

## Supporting information

Supporting Information

Supplemental Video 1

## Data Availability

The data that support the findings of this study are available from the corresponding author upon reasonable request.
