# Supplementary material for: Toward Exotic Layered Materials: 2D Cuprous Iodide
Source: Adv Mater. 2022 Jan 19;34(9):2106922. doi: 10.1002/adma.202106922 (PMC11475451; doi:10.1002/adma.202106922)
Supplement: Supplementary file 1 — Supporting Information [file ADMA-34-2106922-s001.pdf]

ADVANCED MATERIALS

Supporting Information

for *Adv. Mater.,* DOI: 10.1002/adma.202106922

Toward Exotic Layered Materials: 2D Cuprous Iodide

Kimmo Mustonen, Christoph Hofer, Peter Kotrusz, Alexander Markevich, Martin Hulman, Clemens Mangler, Toma Susi, Timothy J. Pennycook, Karol Hricovini, Christine Richter, Jannik C. Meyer, Jani Kotakoski,* and Viera Skákalová**

Supporting Information

Interconnected crystal domains

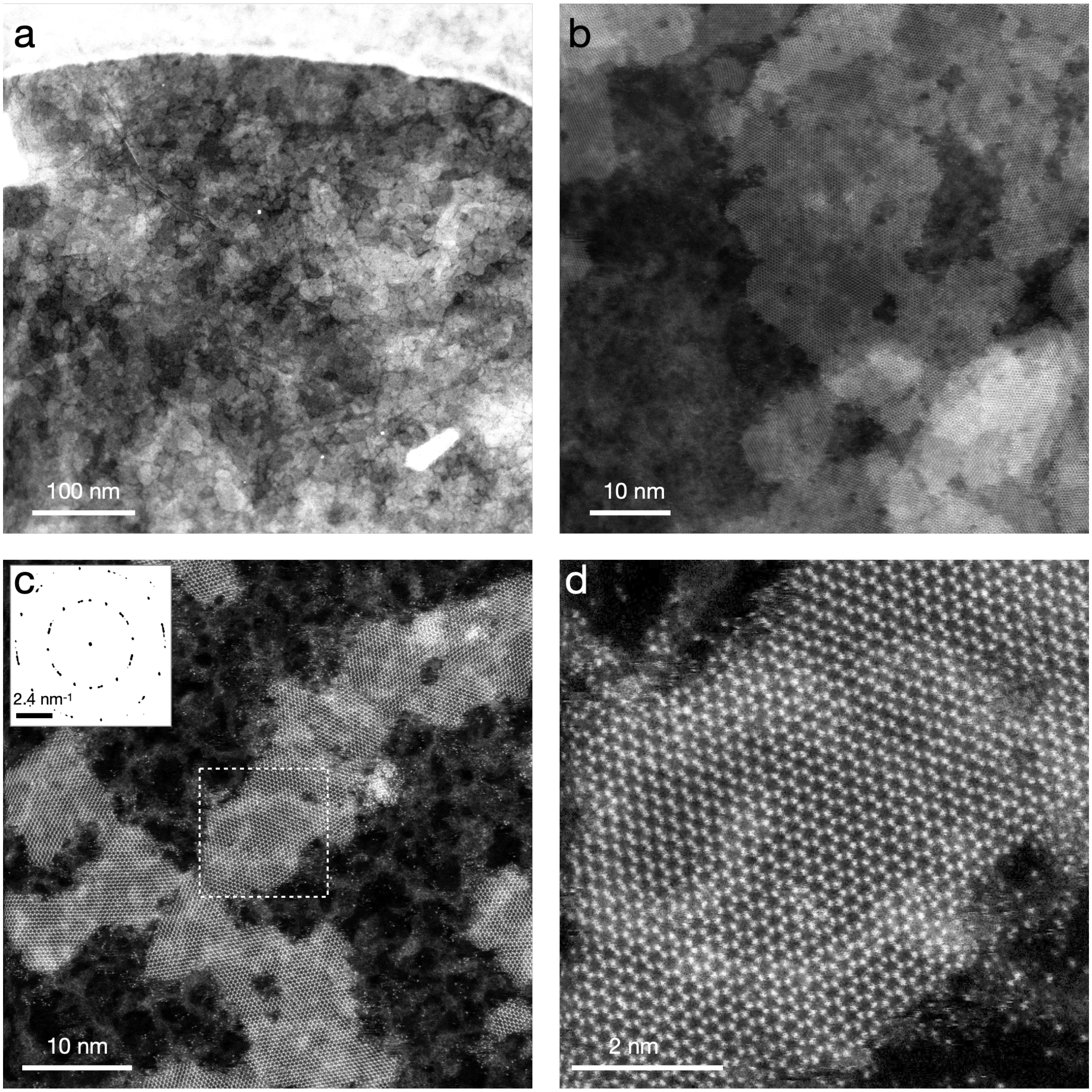

Figure S1: (a-b) Interconnected 2D h-CuI crystal domains in few-layer rGO. (c) 48 nm × 48 nm overview of interconnected domains and a Fourier transform of the image in the inset showing their respective orientations. The rectangle highlights the area from which the real-space ADF STEM tilt-series shown in Figure 4 was acquired. The highlighted area is also magnified in panel (d).

Anisotropy in 2D h-CuI in-plane lattice constant

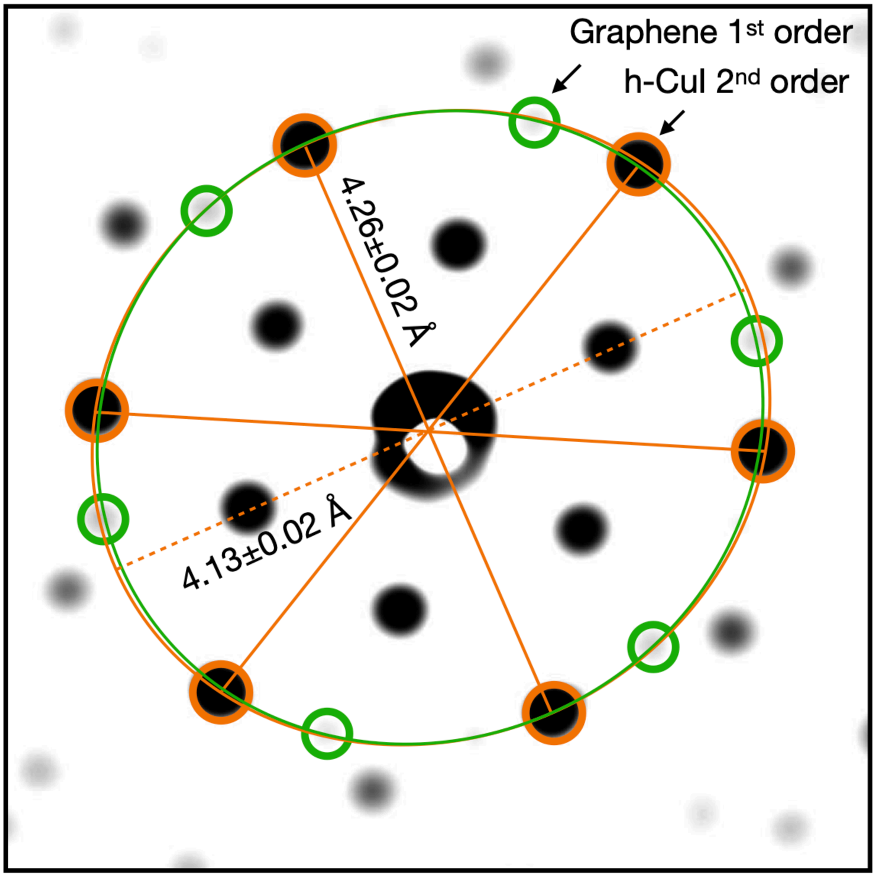

Figure S2: Nano-beam diffraction pattern of a single h-CuI crystal. The graphene 1st order and h-CuI 2nd order Bragg reflections are separately fitted with an elliptical line shape (the patterns are elliptically distorted due to a spurious sample tilt), which allows a direct comparison of the commensurability of the two structures in different directions. The measured lattice parameter values for h-CuI are indicated along the respective directions in the reciprocal space. Note that the lines are drawn only to guide the eye and thus also the d-spacing values tabulated next to them are indicative of distances in real-space in certain directions, and not in the reciprocal space.

2D h-CuI crystal orientation with respect to graphene

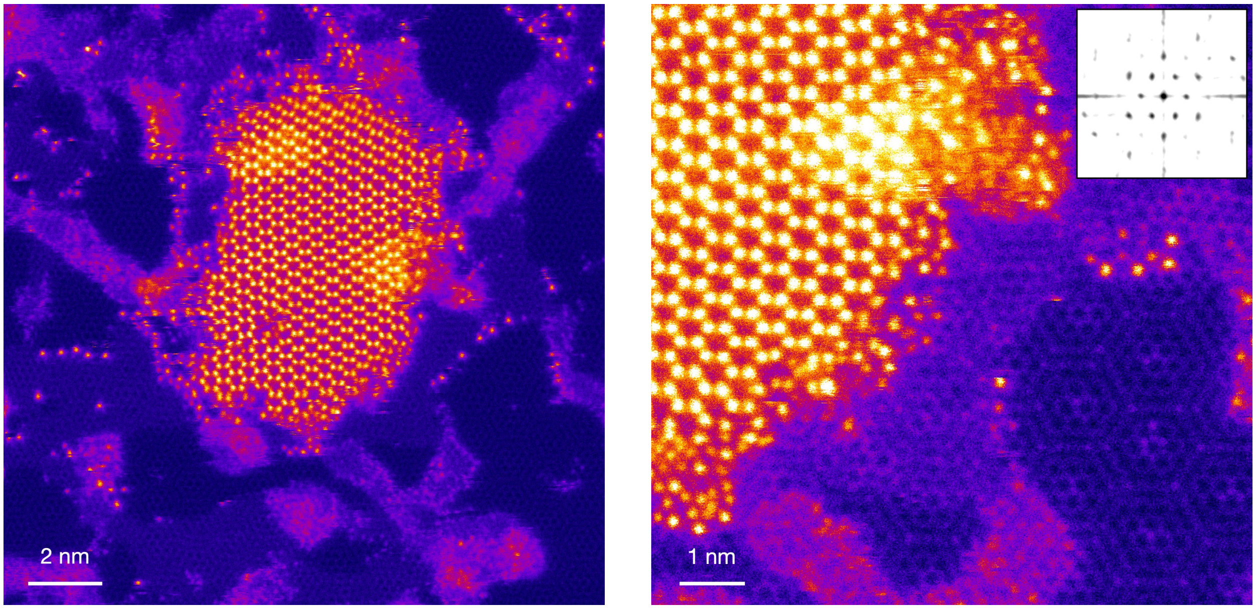

Figure S3: ADF STEM image of a small 2D h-CuI crystal and its edge. Note that the bilayer graphene moiré is visible in the background in the right panel. A Fourier transform of the right panel is shown in the inset.

Cross-sectional imaging of 2D h-CuI

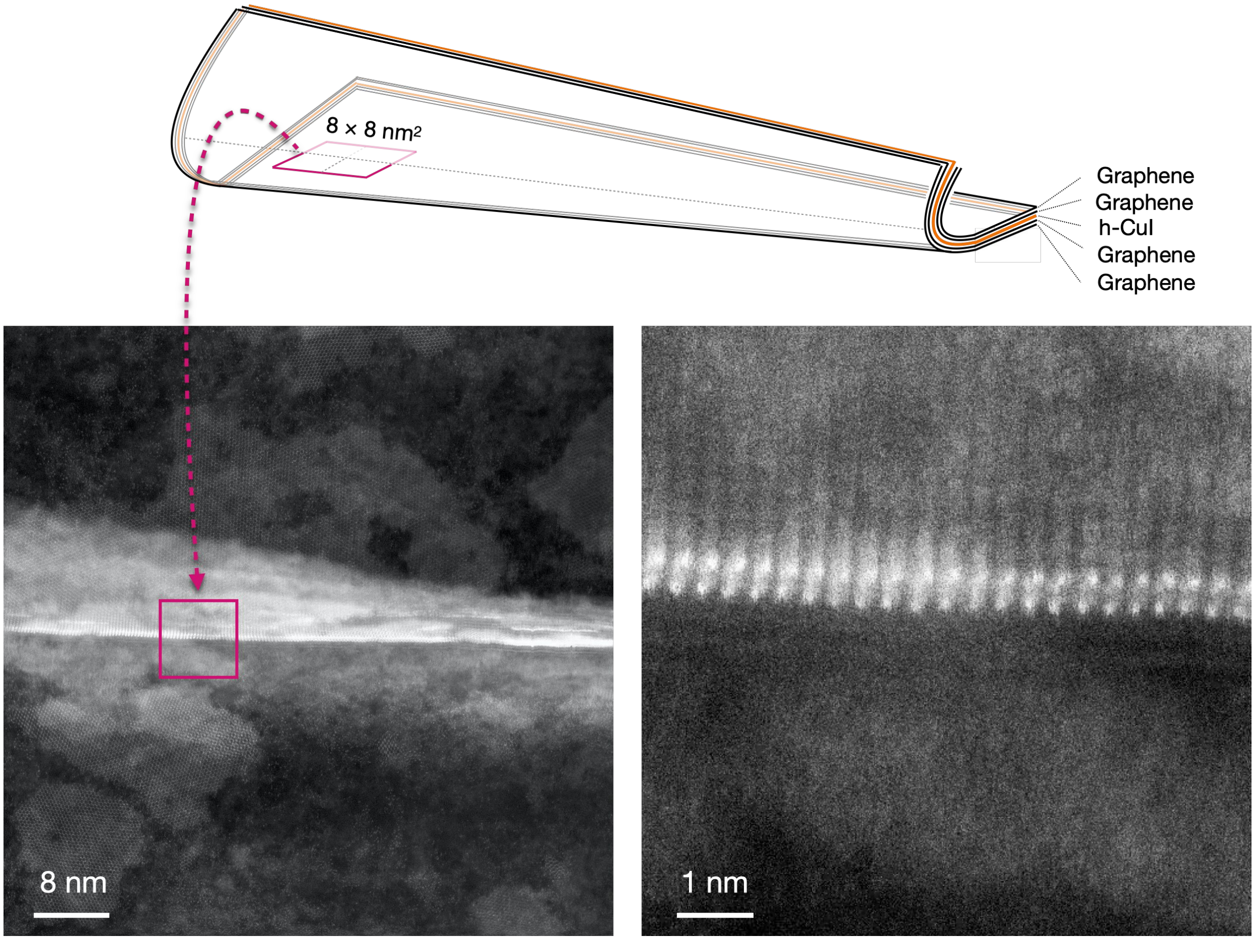

Figure S4: The sample area from which the cross-sectional images shown in Figure 3 were acquired. The top row shows a schematic drawing of the folded multilayer graphene/h-CuI heterostructure.

STEM– and electron diffraction–tilt experiments

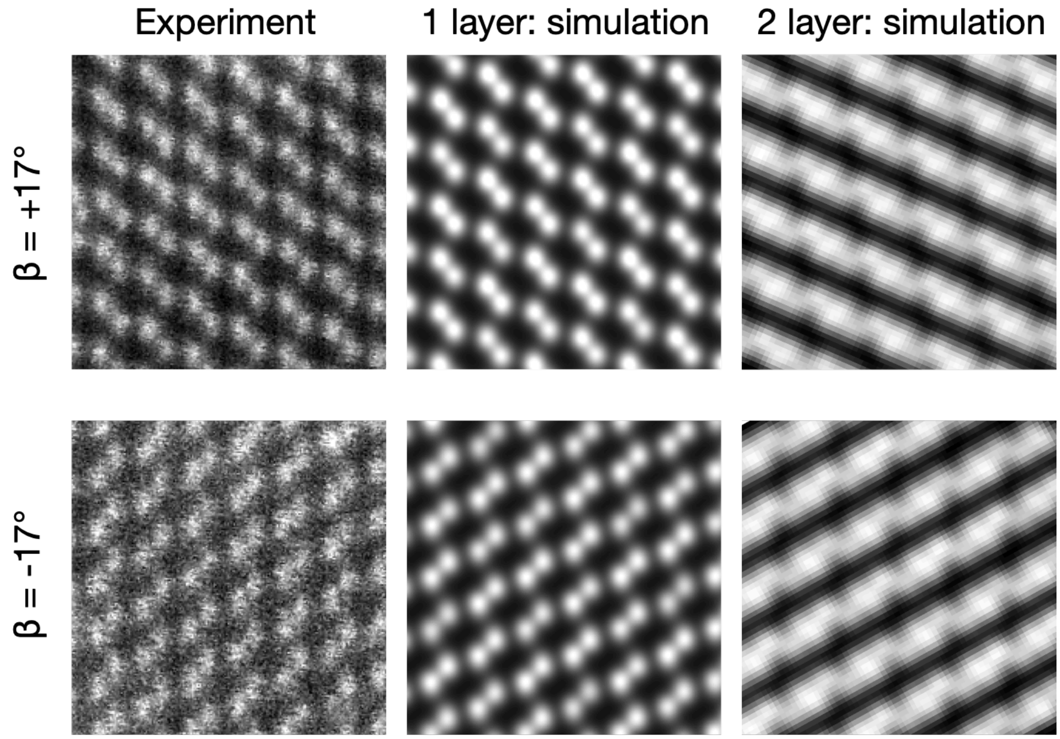

Figure S5: Experimental image of monolayer CuI (left), simulation of monolayer CuI (center) and bilayer CuI (right). The tilt angle is +17° for the top row and -17° for the bottom row images with a horizontal tilt axis with respect to the image plane.

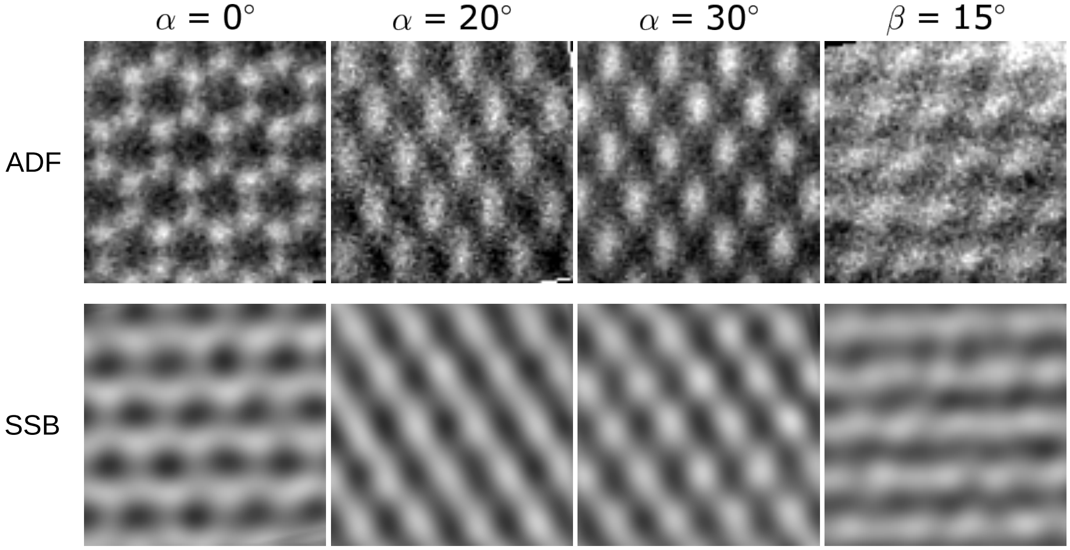

Figure S6: Few-tilt series of h-CuI ADF STEM and reconstructed SSB images used for the 3D reconstructed model in Figure 5 of the main text.

As a further method of confirming the correct identification of β-CuI we probed the diffraction space by nano-beam (NBED) electron diffraction at different tilt angles and compared the experimental intensity of the first order diffraction spots with simulated intensities. For simplicity, we only considered those orientations that are aligned with the tilt axis. As a result of the three-fold symmetry of the crystal, there is a slight break in the inversion symmetry of the diffraction peaks. This asymmetry is also observed in hexagonal boron nitride.[49] The simulated diffraction intensities and experimental intensities (solid line and markers, respectively), are shown in Figure S7 with the tilt axis vertically aligned. Although all six peaks match well with the calculated intensities, small deviations can be explained by the finite accuracy of the tilt angle and the tilt of the structure itself. These mismatches appear more likely at high gradients of the simulated curve (e.g. close to 0°).

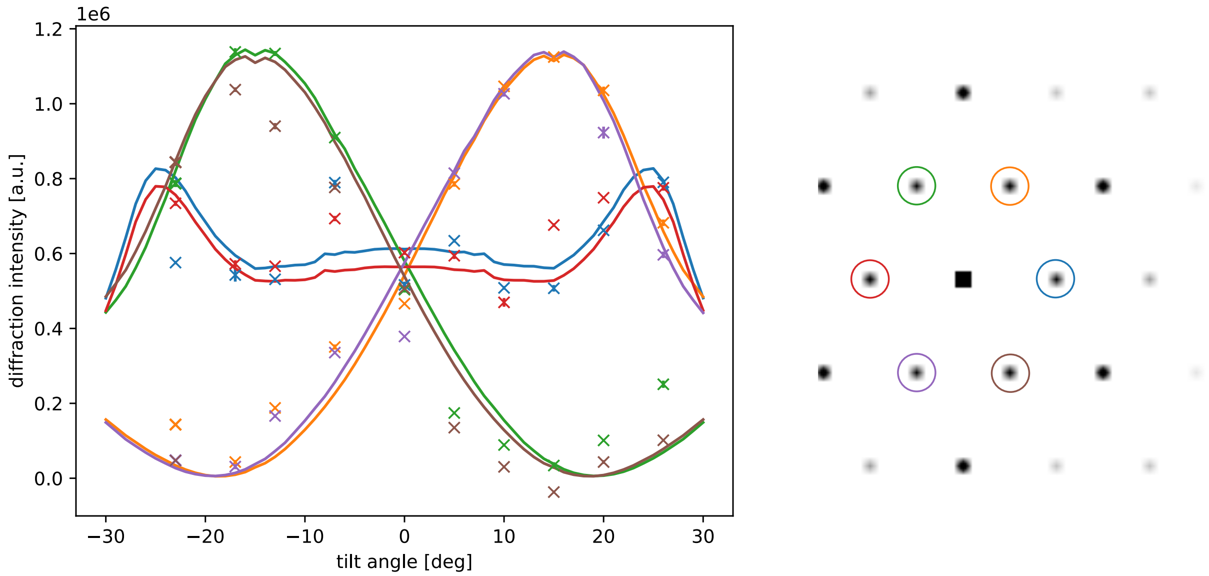

Figure S7: Simulated (solid line) and experimental (crosses) diffraction intensities of all six 1st order Bragg reflections of h-CuI as a function of tilt angle.

Dynamics and stability under electron exposure

HR-TEM image sequences were acquired at 80 keV electron energy to study the dynamics of CuI under the electron beam which gives deeper insights into the overall stability of the structure. Figure S8 shows a three frame sequence under the optimal focus providing the ideal contrast of graphene and h-CuI. The time-resolved sequences demonstrate anisotropic degradation along the zig-zag direction, which can be explained by the release of Cu and I in the different sublattice sites at the crystal edge. While the released I atoms are trapped between the graphene layers and randomly move around, the Cu atoms diffuse outside and react with the residual oxygen gas in the TEM vacuum, forming CuO nanoparticles. The composition of these particles is confirmed by their lattice spacing and elemental composition (cf. Figure S9). Further on, the size of the particles increases along with the greater electron dose, which is a result of the dissociation of further Cu atoms. The blue rectangle in Figure S8 indicates the increasing lateral size of the particles.

In this particular case the 2D h-CuI crystal is in alignment with the graphene sheets, as is indicated by the Fourier transforms below the frames, and small rotations around the ideal alignment are observed. However, also much larger rotations that correspond to invariant translations within the hexagonally symmetric space are possible. The structure observed here, for instance, as soon as the crystal has achieved small enough size, rotates by ca. 30° in the last frame of the series. These discrete orientations of 2D h-CuI with respect to the graphene sheets with small deviations further elaborate the importance of the interaction between the two lattices.

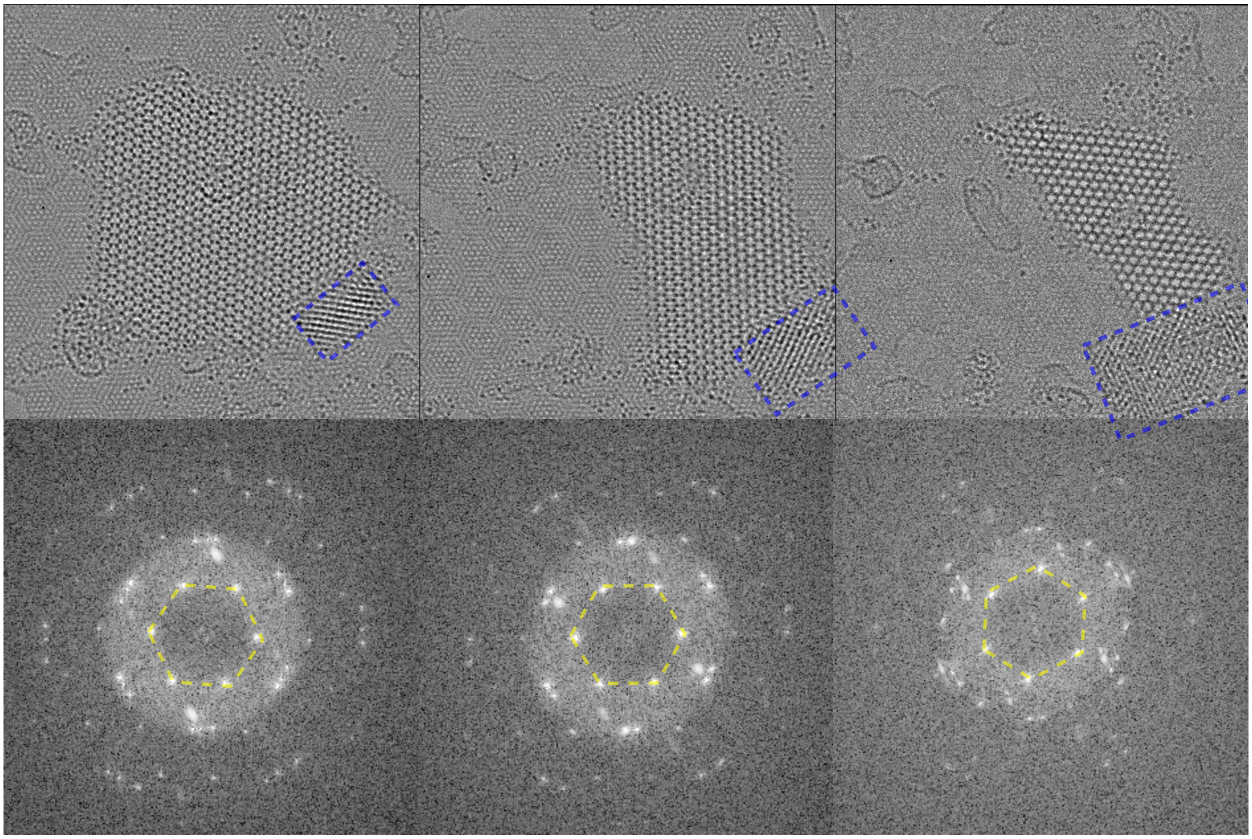

Figure S8: Series of HR-TEM images of h-CuI. The blue rectangle highlights the CuO particle that grows larger frame-by-frame. The images on the bottom row show the corresponding Fourier transforms, highlighting the orientation of h-CuI (yellow broken line).

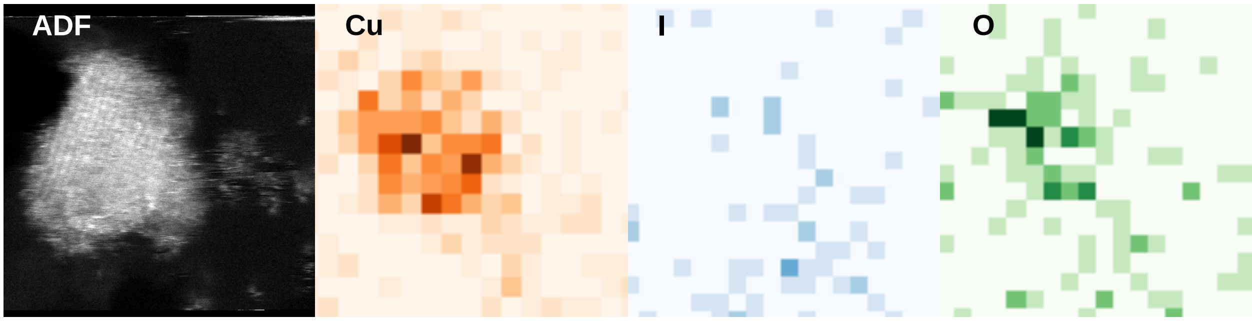

Figure S9: ADF STEM image and energy dispersive x-ray spectroscopy maps of the CuO particle formed after electron irradiation of h-CuI crystal shown in Figure S8.

Density functional theory results

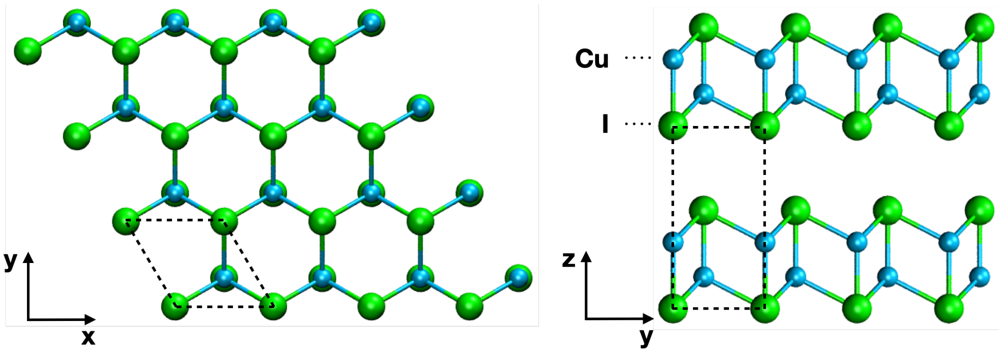

Figure S10: Atomic structure of β-CuI represented by the $4 \times 4 \times 2$ supercell with the primitive cell outlined.

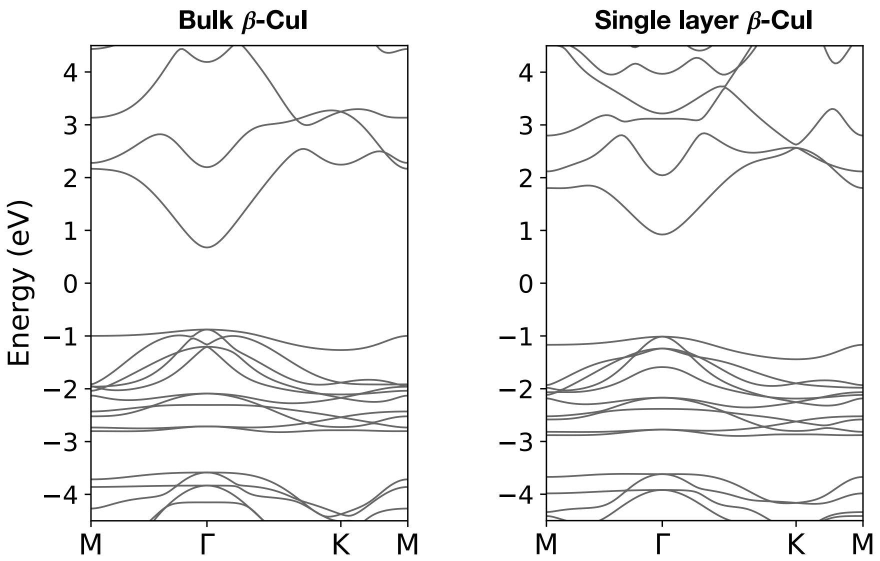

Figure S11: Band structures for the unit cell of bulk and single layer β-CuI calculated with the optPBE-vdW density functional.

Table S1: Lattice constants and interlayer binding energy of bulk β-CuI calculated with the optPBE-vdW density functional.

a, Å	c, Å	E_{bind}, meV/Å^2
4.203	7.345	13.40

Table S2: Band gap in eV for bulk and single layer β-CuI calculated with the optPBE-vdW and HSE06 functionals.

	optPBE-vdW	HSE06
Bulk	1.554	2.683
Single layer	1.936	3.172

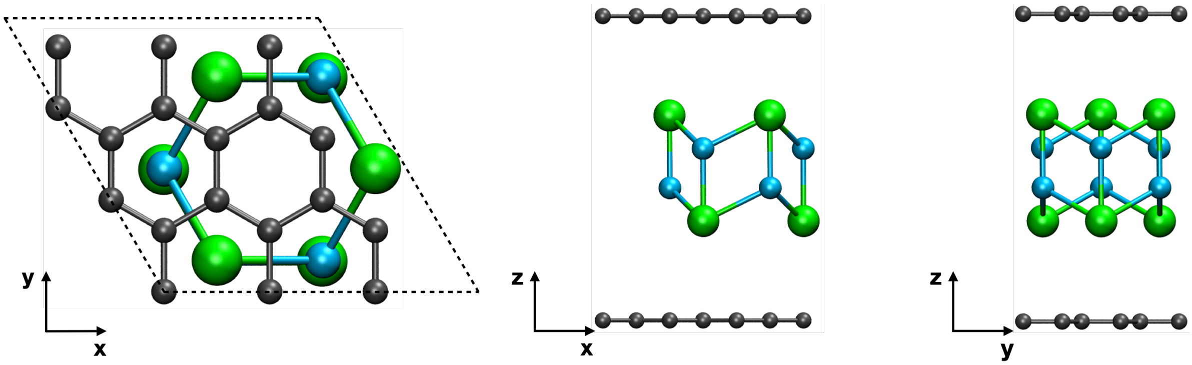

Figure S12: Hexagonal unit cell of h-CuI/graphene heterostructure.

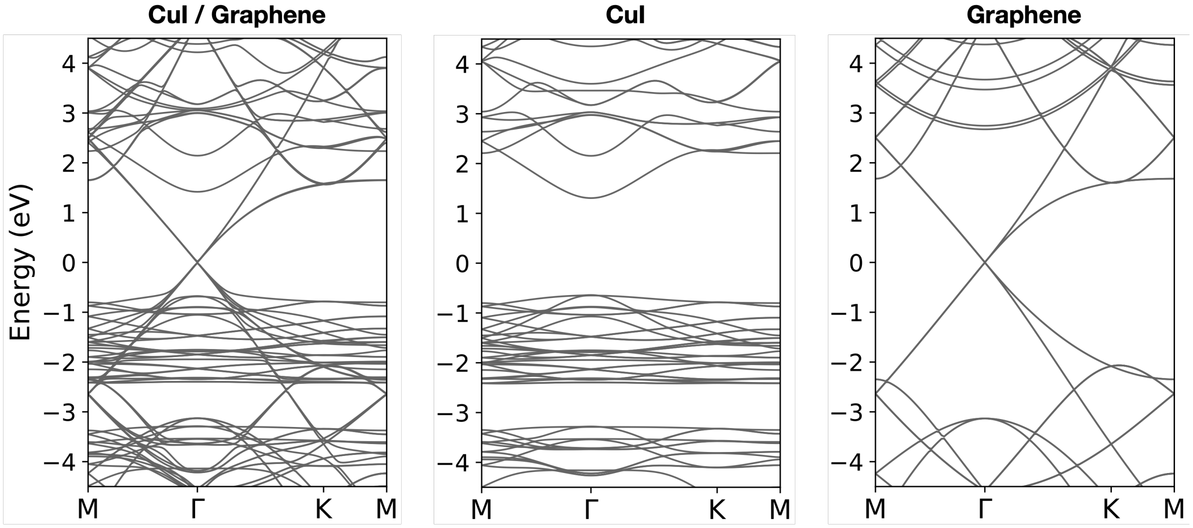

Figure S13: Band structures of h-CuI/graphene heterostructure, 2×2 supercell of h-CuI and 3×3 supercell of graphene calculated with the optPBE-vdW density functional. Zero energy corresponds to the position of the Fermi level.

Table S3: Distances between graphene and CuI, $d_{\text{Gr-CuI}}$, and binding energies per graphene layer, E_{bind}, for different stacking of graphene and CuI layers. H, B, T correspond to configurations where iodine atoms are positioned above (below) the centers of graphene hexagons, middles of the C–C bonds, and C atoms, respectively.

Position	$d_{\text{Gr-CuI}}$, Å	E_{bind}, meV/Å^2
H	3.666	14.18
B	3.679	14.05
T	3.691	14.03

Synthesis of other 2D metal-halides within graphene films

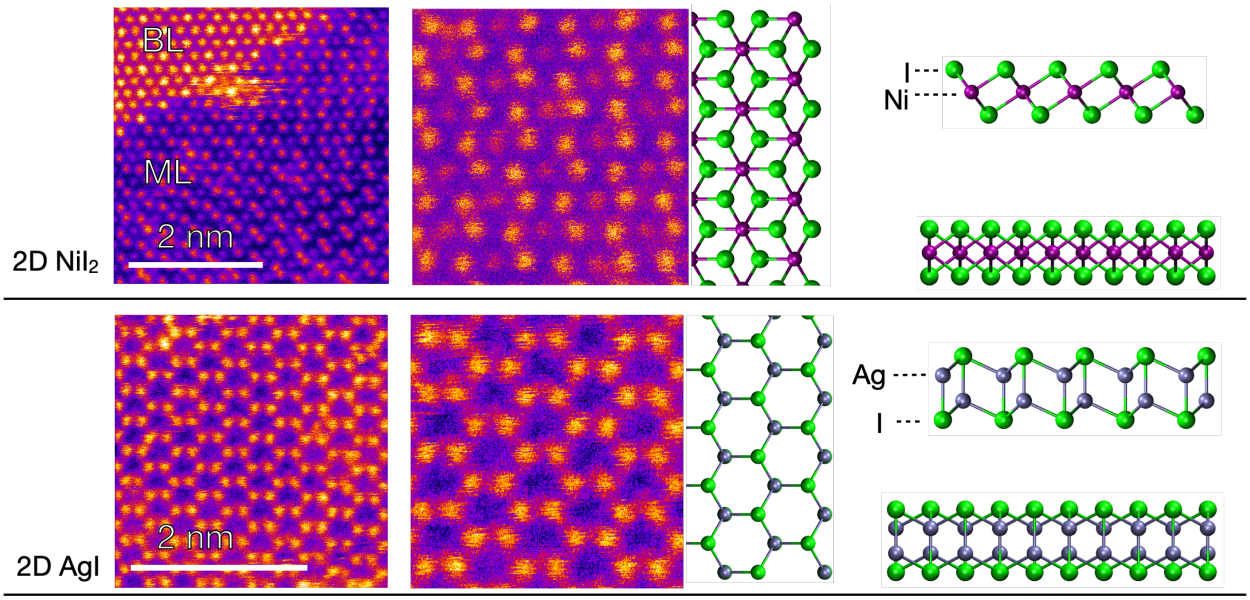

Figure S14: STEM HAADF images of the further synthesized 2D metal-halides: mono- (ML) and bi-layers (BL) NiI_2, and a monolayer of AgI. The images are false-coloured with ImageJ lookup table Fire.
